# Takotsubo cardiomyopathy and transient thyrotoxicosis during combination therapy with interferon-alpha and ribavirin for chronic hepatitis C

**DOI:** 10.1186/1472-6823-14-10

**Published:** 2014-02-03

**Authors:** Carmen Sorina Martin, Luminita Nicoleta Ionescu, Carmen Gabriela Barbu, Anca Elena Sirbu, Ioana Maria Lambrescu, Ioana Smarandita Lacau, Doina Ruxandra Dimulescu, Simona Vasilica Fica

**Affiliations:** 1 Endocrinology Department, Carol Davila University of Medicine and Pharmacy, Elias University Hospital, 17 Marasti Blvd, sector 1, 011461 Bucharest, Romania; 2 Cardiology Department, Elias University Hospital, 17 Marasti Blvd, sector 1, Bucharest, Romania; 3 Endocrinology Department, Elias University Hospital, 17 Marasti Blvd, sector 1, Bucharest, Romania; 4 Radiology Department, Hiperdia, 17 Marasti Blvd, sector 1, Bucharest, Romania; 5 Cardiology Department, Carol Davila University of Medicine and Pharmacy, Elias University Hospital, 17 Marasti Blvd, sector 1, Bucharest, Romania

**Keywords:** Takotsubo cardiomyopathy, Thyrotoxicosis, Chronic hepatitis C, Interferon-alpha, Ribavirin

## Abstract

**Background:**

Thyroid dysfunction is a common complication of chronic hepatitis C (CHC) and its therapy**.** Takotsubo cardiomyopathy (TCM) is a multifactorial, stress related cardiomyopathy, rarely reported in association with thyrotoxicosis. Simultaneous occurrence of TCM and thyrotoxicosis due to hepatitis C and its treatment has never been reported.

**Case presentation:**

A 47-year-old woman was admitted for acute chest pain, dyspnea, palpitations and diaphoresis. She had been diagnosed with CHC and had undergone 7 months of IFNα and Ribavirin therapy. At admission electrocardiogram (ECG) showed ST segment elevation, negative T waves and troponin was elevated suggesting ST segment elevation myocardial infarction (STEMI). Echocardiography demonstrated left ventricular apical akinesia and ballooning, with a left ventricular ejection fraction (LVEF) of 35%. Contrast angiography showed normal epicardial coronaries, yet a ventriculogram revealed left ventricular apical ballooning, consistent with TCM. Cardiac MRI showed left ventricle apical ballooning and no late enhancement suggesting the absence of any edema, scar or fibrosis in the left myocardium. She was diagnosed with non-autoimmune destructive thyroiditis: TSH=0.001 mU/L, free T4=2.41 ng/dl, total T3=199 ng/dl and negative thyroid antibodies. The thyroid ultrasonography showed a diffuse small goiter, no nodules and normal vascularization of the parenchyma. Following supportive treatment she experienced a complete recovery after a few weeks and she successfully completed her antiviral treatment, with no thyroid or cardiovascular dysfunction ever since. In patients treated with IFNα for CHC, the prevalence of thyroid dysfunction varies between 2.5–45.3% of cases. TCM is a stress related cardiomyopathy characterized by elevated cardiac enzymes, normal coronary angiography and an acute, transient, left ventricular apical dysfunction that mimics myocardial infarction. Most of the patients survive the initial acute event, typically recover normal ventricular function within one to four weeks and have a favorable outcome, as was the case with our patient. Thyrotoxicosis induced stress cardiomyopathy is rare and has been mostly reported in association with Graves’ disease, thyroid storm, thyrotoxicosis factitia or following radioiodine therapy for toxic multinodular goiter.

**Conclusion:**

Routine thyroid screening should be done in patients receiving IFN-alpha and Ribavirin for CHC and thyrotoxicosis should be considered as a possible and treatable underlying cause of TCM.

## Background

Every year, 3–4 million people are infected with the hepatitis C virus (HCV) and about 150 million people are nowadays chronically infected worldwide [1]. Thyroid dysfunction represents the most common endocrine manifestation of chronic hepatitis C (CHC). Combination therapy with Interferon-alpha (IFNα) and Ribavirin represents the standard treatment for CHC [2]. In patients treated with IFNα for CHC, the prevalence of thyroid dysfunction varies between 2.5–45.3% of cases [3], depending on the study and diagnostic criteria applied. The main types of interferon induced thyroiditis are: autoimmune thyroiditis (Hashimoto’s thyroiditis, Graves’ disease or the development of thyroid antibodies) and non-autoimmune thyroiditis (destructive thyroiditis or non-autoimmune hypothyroidism) [4]. Furthermore, thyroid dysfunction is more prevalent in patients treated with IFN-α and Ribavirin combination therapy (12.1%) than in patients treated with IFN-α alone (6.6%) [5].

Takotsubo cardiomyopathy (TCM) is a multifactorial, stress related cardiomyopathy characterized by elevated cardiac enzymes, normal coronary angiography and an acute, transient, left ventricular apical dysfunction that mimics myocardial infarction. TCM caused by thyrotoxicosis has previously been reported in about 12 cases since 2004 [6].

We report a rare case of TCM associated with transient thyrotoxicosis, in a female patient treated with IFN-α and Ribavirin for CHC, in order to highlight an unusual complication of thyrotoxicosis and the difficulties associated with the management of CHC.

## Case presentation

**Figure 1:**
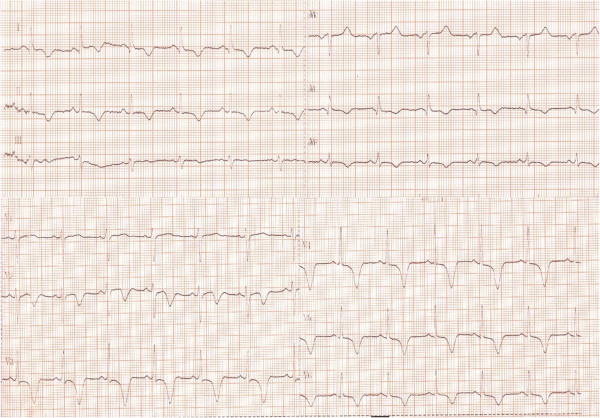
ECG at admission showing sinus rhythm 85 beats/min, ST segment elevation and negative T wave in DI, DII, aVF, aVL, V2-V6 leads.

**Figure 2:**
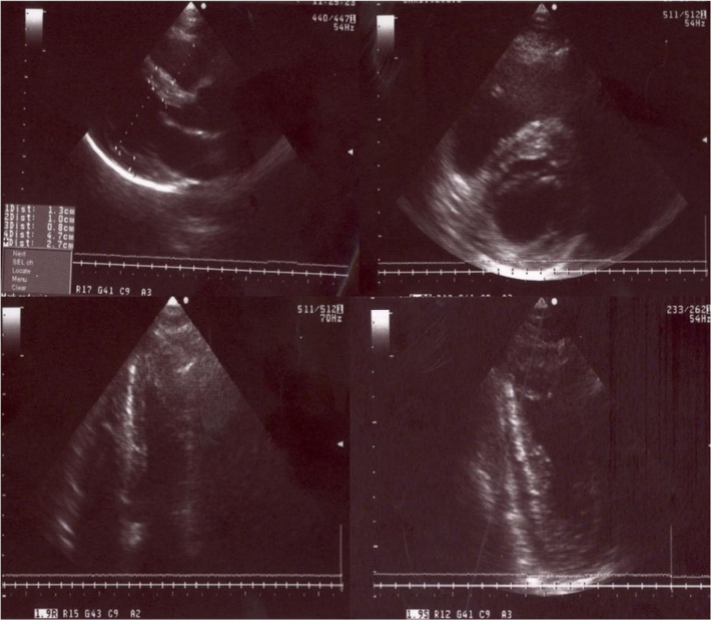
Baseline echocardiography showing normal cardiac cavities, septal hypokinesia, left ventricular apical akinesia and ballooning.

**Figure 3:**
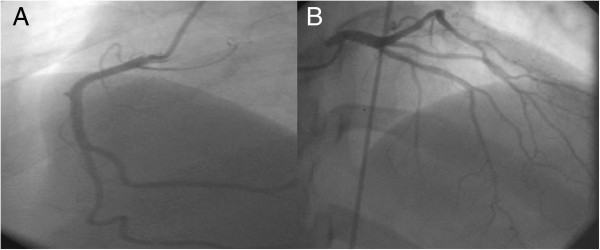
Right (A) and left (B) coronary arteriograms with normal coronary arteries without vasospastic phenomenon.

**Figure 4:**
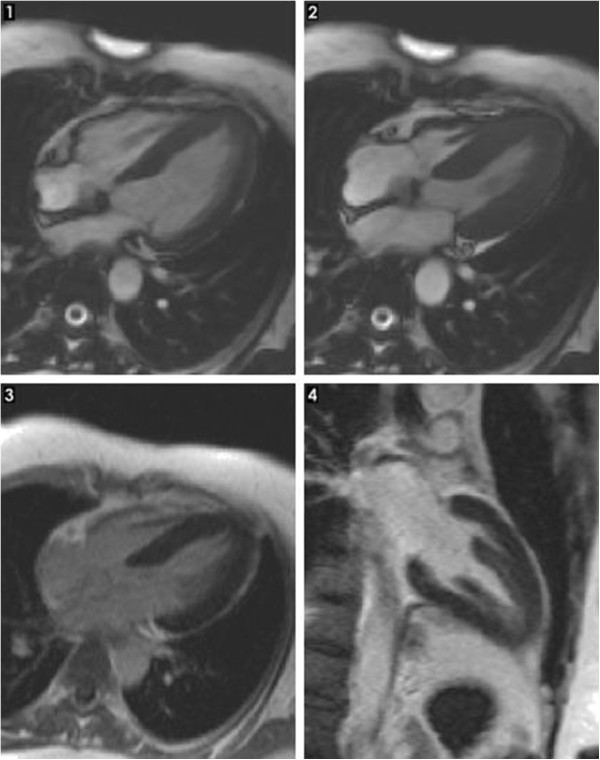
Cardiac MRI: 1,2 cine steady state free precession (SSFP) in four chambers view with apical ballooning; 3,4 triple inversion-recovery post injection of contrast agent with no late enhancement in the left myocardium.

In 2007, a 47- year- old Caucasian woman was admitted for acute chest pain, dyspnea, palpitations and diaphoresis. The symptoms had started 3 days prior to admission, after experiencing emotional stress related to public lecture. Metoprolol 100 mg/day had been started. The patient was a nonsmoker and had no personal history of cardiovascular disorders. She had been diagnosed with CHC in 2006 and had undergone 7 months of pegylate-IFN-alpha (180 μg/week) and Ribavirin (1000 mg/day) therapy. Physical examination on admission revealed a heart rate of 85 beats/min and blood pressure of 160/80 mmHg. Routine biochemical and hematological tests were normal, except for the presence of anemia (hemoglobin = 9.74 g/dl, hematocrit = 31.4%), leucopenia (white blood cell count = 4290/mm3, neutrophils = 2250/mm3), hypocholesterolemia (123 mg/dl) and mild increase in liver enzymes (ALT = 53, AST = 55UI/L). C reactive protein was negative. Plasma free metanephrines = 68 pg/ml (<90) and normetanephrines = 126 pg/ml (<180) were in the normal range. ECG at admission showed sinus rhythm 85 beats/ min, ST segment elevation and negative T waves in DI, DII, aVF, aVL, V2-V6 leads (Figure 1). The peak troponin was 2 ng/ml (<0.29). Echocardiography showed normal cardiac cavities, left ventricular apical akinesia and ballooning, septal hypokinesia and a LVEF of 35% (Figure 2). Contrast angiography showed normal epicardial coronaries (Figure 3), yet a ventriculogram revealed left ventricular apical hypokinesis and ballooning, consistent with TCM. For differential diagnosis and tissue characterization, cardiac MRI was performed, showing apical ballooning of the left ventricle. There were no bright areas in T2 sequence with fat suppression and no late enhancement suggesting the absence of any edema, scar or fibrosis in the left myocardium (Figure 4).

During the first days the patient received supportive therapy with angiotensin converting enzyme inhibitors, beta-blockers (metoprolol 200 mg/day), platelet aggregation inhibitors, statin, low molecular weight heparin, nitrates and anxiolytics, with favorable resolution of dyspnea and no chest pain. The troponin returned rapidly to normal within 72 hours (0.044 ng/ml). Despite high dose beta-blockers she was persistently tachycardic. The endocrinological evaluation highlighted: patient with no family or personal history of thyroid disorders, without fever, pain in the cervical region or ophthalmopathy; she had palpitations, excessive sweating, intolerance to heat, nervousness, easy fatigability, hyperkinesia; TSH = 0.001 mU/L, free T4 = 2.41 ng/dl (0.71-1.85), total T3 = 199 ng/dl (81–178), anti-thyroid peroxidase antibodies (TPOAb) = 3.8 IU/ml (0–12), antithyroglobulin antibodies = 56 UI/ml (<115) and anti-thyrotropin receptor antibodies (TRAb) = 1.05 IU/L (<1.75). The thyroid ultrasonography showed a diffuse heterogenic small goiter, no nodules and normal vascularization of the parenchyma. Non-autoimmune destructive thyroiditis was diagnosed and the patient continued treatment with beta-blockers and anxiolytics for the transient thyrotoxic phase of the thyroiditis. Antiviral treatment for CHC was stopped about 4 weeks and afterwards resumed, with the same dosages, for another 11 month.

Dynamic ECG over the first 10 days revealed regression and disappearance of the ST segment elevation, with persistently negative T waves, which normalized 2 weeks later. Repeat echocardiography performed 2 weeks after the admission showed a normal LVEF, with apical hypokinesia and 4 weeks later showed complete recovery of the apical akinesia. The clinical picture with the absence of late angina, the normalization of the ECG, associated with normal epicardial coronaries at angiography and the transient, acute, left ventricular apical ballooning, enabled us to diagnose a stress-related, reversible, ventricular apical dysfunction.

**Figure 5:**
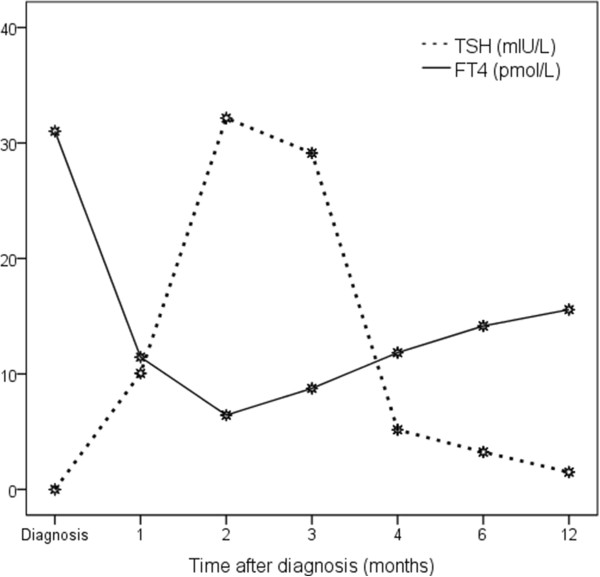
Thyroid function tests in the first year following the diagnosis.

In the next 3 months she developed transient hypothyroidism and then became euthyroid (Figure 5). She successfully completed her antiviral treatment and she continues to be monitored as an outpatient with thyroid function tests and cardiac echocardiography every 6 month, with no thyroid or cardiovascular dysfunction ever since.

## Discussion

TCM is an acute impairment of the cardiac function, named after the Japanese fishing pot used for trapping octopus, because of the peculiar left ventricle apical ballooning on left ventriculogram. The modified Mayo Clinic criteria for the diagnosis of TCM are: 1) transient hypokinesis, dyskinesis or akinesis of the left ventricular midsegments, with or without apical involvement; the regional wall-motion abnormalities extend beyond a single epicardial vascular distribution, and a stressful trigger is often, but not always, present; 2) absence of obstructive coronary disease or angiographic evidence of acute plaque rupture; 3) new electrocardiographic abnormalities (either ST-segment elevation and/or T-wave inversion) or modest elevation in cardiac troponin level; 4) absence of pheochromocytoma or myocarditis [7].

Our case is a typical non-autoimmune destructive thyroiditis, in a woman with no personal history of thyroid or cardiovascular disorders, but with known history of CHC for which she was receiving IFNα and Ribavirin therapy for 7 months prior to the diagnosis. A higher prevalence of thyroid disorders has been reported in HCV-infected patients than in the general population [8]. Antiviral therapy of CHC possibly induces de novo or exacerbates pre-existing silent thyroid disorders [9]. Thyroid diseases may occur anytime during therapy and are not absolute contraindications for IFNα or Ribavirin therapy. Furthermore, IFN-α dosage or treatment duration and the virological response to the treatment with IFN-α and Ribavirin seem not to be related to the incidence of thyroid dysfunction [10].

This case is special because of the unusual concomitant association of two transient illnesses: IFN-α induced thyroiditis and acute left ventricular apical ballooning. The association between thyrotoxicosis and cardiac complications is well known, yet thyrotoxicosis induced stress cardiomyopathy is rare [11]. TCM was reported in association with varied thyroid functional status, ranging from severe hypothyroidism [12], apathetic [13] or subclinical hyperthyroidism [14], endogenous or exogenous thyrotoxicosis [15,16], transient hyperthyroidism [17], thyroid storm [11,18] to even normal thyroid function [19]. TCM was also reported following radioiodine therapy [20] or surgical treatment for thyroid disorders [21]. To the best of our knowledge, until now, thyrotoxicosis induced stress cardiomyopathy has never been reported in association with thyroiditis precipitated by IFNα and Ribavirin therapy for CHC.

The patient was initially admitted for symptoms suggesting STEMI. However, echocardiography showing left ventricle apical ballooning, correlated to the coronary angiography that revealed no significant blockage and the absence of late enhancement on cardiac MRI, enabled us to diagnose a stress related reversible ventricular apical dysfunction. Stress-induced cardiomyopathy is an increasingly reported syndrome and it may account for approximately 2% of suspected acute coronary syndromes [22]. Left ventriculography is perhaps the best imaging modality to demonstrate the pathognomonic wall motion disturbance and to evaluate LVEF [23]. Cardiac MRI is, also, an important diagnostic modality for TCM, that can evaluate the regional wall motion abnormalities, can quantify ventricular function and identify the presence of edema/inflammation and the absence of necrosis/fibrosis [24]. Furthermore, cardiac MRI can differentiate TCM, characterized by the absence of delayed gadolinium hyperenhancement, from myocardial infarction and myocarditis, in which the opposite occurs. Both left ventriculography and cardiac MRI supported the TCM diagnosis in our case. Yet, myocarditis could not be ruled out by immunohistological examination of endomyocardial biopsies (EMB) or virus PCR, as proposed by the fourth Mayo criteria. However, in the reported case series of TCM, endomyocardial biopsy, the gold standard diagnostic for myocarditis, has not been systematically performed [25]. In a recent review, which included 286 patients from 14 studies, only 15 patients from 4 studies underwent EMB, with no evidence of myocarditis in any of them [26].

Postulated mechanisms for TCM pathogenesis include: direct cardiotoxicity of catecholamine excess, epicardial coronary vasospasm, micro-vascular dysfunction, in situ coronary thromboembolus with complete later recanalization, shifts in cardiac metabolism from fatty acids towards carbohydrates and left ventricular outflow tract obstruction, resulting in myocardial stunning [27]. Inflammation and oxidative stress might also play a role [28].

75% of patients with TCM have elevated serum catecholamine levels, even higher than patients with STEMI [29], which may explain the hypothesis of vascular dysfunction due to micro-vascular spasm. Supraphysiologic levels of catecholamines could trigger a switch in the coupling of β-2 adrenoreceptors from Gs to Gi protein, leading to decreased contractility. The left ventricular apex may be more vulnerable to catecholamine excess due to greater β-adrenergic receptor density and/or increased myocardial responsiveness [30]. This catecholamine hypotesis is also supported by the rat models, where Takostubo has been prevented with adrenergic blockade, by sampling of cardiac catecholamine levels in TCM [31] and by the reported cases in association with pheochromocytoma [32] and use of exogenous sympathomimetics.

The cause of myocardial stunning in thyrotoxic patients with normal coronary arteries is still unknown. Thyroid hormones have both direct and indirect actions on the cardiovascular system, can alter cardiovascular hemodynamics and represent a cause of cardiomyopathy. In thyrotoxic patients myocardial ischemia may occur due to coronary vasospasm [33], in situ coronary thromboembolus or direct metabolic effects of thyroid hormones.

The thyroid and adrenergic axes are closely interrelated, and pathologically high levels of thyroid hormones cause exaggerated chronotropic and contractile responses to catecholamines. Thyroid hormones modulate the transcription of multiple genes and also have extranuclear actions in cardiac myocytes, leading to various cardiovascular effects, similar to catecholamine-mediated stimulation of β-adrenergic receptors [34]. Thyroid hormones upregulate β-adrenergic receptors in many tissues, including the heart [35], thus, increasing sensitivity to catecholamines and potentiating catecholamine action. Furthermore, experimental animal data have shown that cardiovascular responses to hyperthyroidism are preserved in mice lacking all three β adrenergic receptors compared with wild-type mice [36]. An additional direct action of thyroid hormones at the intracellular level has been suggested based on the finding of thyroid hormone receptor expression on cardiac myocytes [37]. Hyperthyroidism can also mimic a state of catecholamine excess, mediated through a change in the balance between sympathetic and vagal innervation [38].

On the other hand, some studies have shown that plasma catecholamine levels, as in our case, are usually normal in TCM [6] and that the increase in β-adrenergic receptors is not always accompanied by a corresponding increase in cardiovascular sensitivity to catecholamines [39,40]. Some authors have even suggested that TCM might not be related to thyrotoxicosis, per se, but it could be a specific complication of autoimmune thyrotoxicosis [41]. Yet, the publication of TCM associated with exogenous thyrotoxicosis and radioiodine-induced thyroiditis argues against this theory.

Anyway, even if catecholamine levels are not increased in hyperthyroidism, their action seems to be amplified resulting in a hyper-responsive state to a minimal stimulation. Thyroid hormones can induce a catecholamine-mediated cardiotoxicity, which remains the most commonly proposed mechanism in TCM.

Despite the potential severe presentation, most of the patients survive the initial acute event, typically recover normal ventricular function within one to four weeks and have a favorable outcome, as was the case with our patient. Since TCM usually progresses to a full recovery, the question of whether patients with hyperthyroidism induced TCM would have recovered even without the correction of the thyroid function remains to be answered. Although infrequent, recurrence of the syndrome has been reported and seems to depend on the nature of the trigger [42]. Our patient has been followed for nearly 5 and a half years, with no recurrence of the TCM nor the thyroid dysfunction.

## Conclusion

As previously suggested [4], we believe that TSH and thyroid antibody levels should be measured in all hepatitis C patients prior to starting IFNα therapy and TSH levels should be monitored every three months until completion of IFNα course. In hepatitis C patients treated with IFNα, multidisciplinary teams (gastroenterologist, endocrinologist, cardiologist) should be aware that thyrotoxicosis could be a possible and treatable underlying cause of TCM.

## Consent

Written informed consent was obtained from the patient for publication of this Case report and any accompanying images. A copy of the written consent is available for review by the Editor of this journal.

## Abbreviations

TCM: Takotsubo cardiomyopathy; IFN α: Interferon-alpha; CHC: Chronic hepatitis C; HCV: Hepatitis C virus; ECG: Electrocardiogram; STEMI: ST segment elevation myocardial infarction; LVEF: Left ventricular ejection fraction; TSH: Thyroid stimulating hormone; TPOAb: Anti-thyroid peroxidase antibodies; TRAb: Anti-thyrotropin receptor antibodies.

## Competing interests

The authors declare that they have no competing interests.

## Authors’ contributions

CSM led the composition of this case report, acquisition of data and drafted the manuscript. IML performed literature review. CGB and AES participated in the endocrinological management and reviewed the manuscript. LNI participated in the cardiological treatment and performed echocardiography interpretation. ISL performed radiological interpretation. DRD and CSM continue clinical follow up of the patient to date. DRD and SVF critically reviewed the manuscript. All authors read and approved the manuscript.

## Pre-publication history

The pre-publication history for this paper can be accessed here:

http://www.biomedcentral.com/1472-6823/14/10/prepub

## Notes

Part of this report was presented as poster at the 34th Annual Meeting of the European Thyroid Association, Lisbon, Portugal, 2009: “Thyrotoxicosis and concomitant Tako-Tsubo cardiomyopathy in a patient with chronic hepatitis C: an unusual clinical association”- Sorina Martin, Simona Fica, Doina Dimulescu, Luminita Ionescu, Mircea Diculescu, Smaranda Lacau, Rodica Rotaru, Dan Deleanu –poster 237, 34th Annual Meeting of the European Thyroid Association, Lisbon, 5–9 Sept. 2009, Acta Medica Portuguesa, 2009, vol.22, p.492, e-ISSN 1646–0758.
